# A proof-of-concept study of growth hormone in children with Phelan–McDermid syndrome

**DOI:** 10.1186/s13229-022-00485-7

**Published:** 2022-01-29

**Authors:** S. Sethuram, T. Levy, J. Foss-Feig, D. Halpern, S. Sandin, P. M. Siper, H. Walker, J. D. Buxbaum, R. Rapaport, A. Kolevzon

**Affiliations:** 1 Department of Pediatrics, Saint Peter’s University Hospital, New Brunswick, NJ USA; 2 Seaver Autism Center for Research and Treatment, Icahn School of Medicine at Mount Sinai, One Gustave L. Levy Place, Box 1230, New York, NY 10029 USA; 3 Department of Psychiatry, Icahn School of Medicine at Mount Sinai, New York, NY USA; 4 Present Address: Mindich Child Health and Development Institute, Icahn School of Medicine at Mount Sinai, New York, NY 10029 USA; 5 Department of Medical Epidemiology and Biostatistics, Karolinska Institutet, Stockholm, Sweden; 6 Department of Neuroscience, Icahn School of Medicine at Mount Sinai, New York, NY USA; 7 Department of Genetics and Genomic Medicine, Icahn School of Medicine at Mount Sinai, New York, NY USA; 8 Department of Pediatrics, Icahn School of Medicine at Mount Sinai, New York, NY USA

**Keywords:** Phelan–McDermid syndrome, PMS, Shank3, Autism spectrum disorder, ASD, Growth hormone, Insulin-like growth factor-1, IGF-1

## Abstract

**Background:**

Phelan–McDermid syndrome (PMS) is caused by 22q13 deletions including *SHANK3* or pathogenic sequence variants in *SHANK3* and is among the more common rare genetic findings in autism spectrum disorder (ASD). *SHANK3* is critical for synaptic function, and preclinical and clinical studies suggest that insulin-like growth factor-1 (IGF-1) can reverse a range of deficits in PMS. IGF-1 release is stimulated by growth hormone secretion from the anterior pituitary gland, and this study sought to assess the feasibility of increasing IGF-1 levels through recombinant human growth hormone (rhGH) treatment, in addition to establishing safety and exploring efficacy of rhGH in children with PMS.

**Methods:**

rhGH was administered once daily for 12 weeks to six children with PMS using an open-label design. IGF-1 levels, safety, and efficacy assessments were measured every 4 weeks throughout the study.

**Results:**

rhGH administration increased levels of IGF-1 by at least 2 standard deviations and was well tolerated without serious adverse events. rhGH treatment was also associated with clinical improvement in social withdrawal, hyperactivity, and sensory symptoms.

**Limitations:**

Results should be interpreted with caution given the small sample size and lack of a placebo control.

**Conclusions:**

Overall, findings are promising and indicate the need for larger studies with rhGH in PMS.

*Trial registration* NCT04003207. Registered July 1, 2019, https://clinicaltrials.gov/ct2/show/NCT04003207.

## Introduction

Phelan–McDermid syndrome (PMS) is caused by deletions in the long arm of chromosome 22 which include the *SHANK3* gene (MIM: 606230), or by pathogenic sequence variants in *SHANK3* [1–4]. PMS is associated with developmental delays, intellectual disability, and autism spectrum disorder (ASD), in addition to renal, cardiac, and gastrointestinal abnormalities, hypotonia, and dysmorphic features [5]. *SHANK3* has been established as the critical gene in PMS [1–4, 6] and appears to account for ~ 0.5% of ASD [7]. *SHANK3* encodes for a master scaffolding protein in the post-synaptic density of excitatory synapses and is responsible for the formation and maintenance of synapses [8]. As such, *SHANK3* and associated pathways represent important targets for intervention.

Evidence from both preclinical and clinical studies suggests that insulin-like growth factor-1 (IGF-1) can reverse deficits in synaptic plasticity and motor learning in mouse and human neuronal models of PMS [9, 10]. A clinical trial with IGF-1 in children with PMS also showed improvement in social withdrawal and restricted behaviors, both core features of ASD [11]. Additional evidence of the utility of IGF-1 comes from animal, human, and human neuronal studies of Rett syndrome, another rare genetic disorder associated with ASD, where IGF-1 was effective in reversing phenotypic features [12–16].

IGF-1 is released mainly by the liver upon growth hormone stimulation and enters the brain from the circulation to promote brain vessel growth [17], neurogenesis, and synaptogenesis [18]. Once IGF-1 binds to the IGF-1 receptor, activation of the PI3K/mTOR/AKT1 and MAPK/ERK pathways induces its downstream effects [19]. Treatment with IGF-1 is generally administered twice daily via subcutaneous injection and requires careful monitoring due to numerous risks, including hypoglycemia. Further, IGF-1 is challenging to manufacture and while commercially approved for short stature due to primary IGF-1 deficiency, it is costly and not readily available. However, IGF-1 levels can be increased intrinsically by growth hormone [20] without the risk of hypoglycemia. Recombinant human growth hormone (rhGH) has an excellent safety profile and approved indications in pediatric and adult populations. One recent case report also supports the use of rhGH in PMS [21]. For these reasons, rhGH was chosen for this trial with the primary aims of demonstrating the feasibility of increasing IGF-1 levels in the blood and establishing safety in PMS. Furthermore, we sought to explore signals of efficacy using a battery of clinical outcome assessments, including the Aberrant Behavior Checklist—Social Withdrawal subscale (ABC-SW) [22] as the primary clinical outcome. The ABC-SW subscale was chosen based on results from the previous clinical trial with IGF-1 in PMS [11].

## Methods

This study was approved by the Program for the Protection of Human Subjects at the Icahn School of Medicine at Mount Sinai, and all caregivers provided written informed consent.

### Inclusion/exclusion criteria

Participants were required to have a confirmed genetic diagnosis of PMS and be between 2 and 12 years of age. Participants were excluded if they had closed epiphyses, active or suspected neoplasia, intracranial hypertension, hepatic insufficiency, renal insufficiency, cardiomegaly/valvulopathy, or allergy to growth hormone or any component of the formulation.

### Drug administration

rhGH was administered in its commercially available form as somatropin (Zomacton). Caregivers were trained by a pediatric endocrinologist (Sethuram, S) to administer rhGH subcutaneously, through demonstration and written material. rhGH was given once daily for 12 weeks using an open-label design. Doses were based on standard clinical practice for children who are not growth hormone deficient with a target dose of 0.3 mg/kg/week. All participants were initiated on half the target dose (0.14–0.16 mg/kg/week) for two weeks as a safety precaution and then increased to a full dose for the remaining 10 weeks. IGF-1 levels were measured every 4 weeks, and IGF-1 *Z* scores were used to guide titration of rhGH dose using two standard deviations (SD) above the population mean as the target.

### Safety measures and laboratories

Medical and psychiatric history was collected prior to the onset of the trial. Safety laboratories, physical examinations, and IGF-1 values were collected at the baseline visit and at each follow-up visit: weeks 4, 8, and 12. Adverse events were collected at every visit using the Systematic Longitudinal Adverse Events Scale (SLAES).

### Clinical measures

The primary clinical outcome was the ABC-SW subscale (ABC-SW) [22]. Additional clinical outcome assessments were used to capture a range of ASD-related symptoms, including the Repetitive Behavior Scales—Revised (RBS-R) [23], the Sensory Profile (SP) [24], other ABC subscales (Table 2), and the Clinical Global Impression—Improvement scale (CGI-I) [25].

### Statistical analyses

Nonparametric Wilcoxon signed-rank tests were used to evaluate differences in clinical outcomes between baseline and week 12. All tests of statistical hypotheses were done on the two-sided 5% level of significance. We selected a single primary efficacy variable (ABC-SW) a priori and did not adjust for multiplicity of statistical tests. All raw *p* values are presented to allow an adjustment post hoc (Table 3). In the case of missing data, we used the last observation carried forward. The sample size was not based on statistical criteria and was determined by feasiblity for this pilot study.

## Results

### Participants

This trial was conducted from September 2019 to June 2020 and terminated early due to COVID-19; the original recruitment target was 10 participants. Six participants were screened, and all met inclusion criteria and were enrolled. Participants (2 males; 4 females) were between 3.2 and 11.4 years of age (7.5 ± 3.2). All participants except one female were pre-pubertal. The one child who was pubertal on physical and biochemical evaluation did not reach menarche. At baseline, all children were of average weight (− 0.85 ± 1.15 SD), height (− 1.38 ± 0.75 SD), and body mass index (-0.82 ± 1.27). All bone ages were within the normal range. Baseline IGF-1 *Z* scores varied between − 1.2 and 2.3 (Table 1).

**Table 1:** rhGH dose in mg/kg/week and IGF-1 *Z* scores

Participant		Baseline	Week 2	Week 4	Week 8	Week 12
1	IGF-1 *Z* score	0.8	–	2.6	4.8	2.2
	rhGH dose	0.15	0.3	0.28	0.24^a^	–
2	IGF-1 *Z* score	1.0	–	6.0	5.0	3.9
	rhGH dose	0.15	0.28	0.24^a^	0.16^a^	–
3	IGF-1 *Z* score	1.1	–	2.8	4.7	1.7
	rhGH dose	0.14	0.3	0.29	0.24^a^	–
4	IGF-1 *Z* score	0.9	–	4.5	1.8	2.9
	rhGH dose	0.16	0.29	0.21^a^	0.19^b^	–
5	IGF-1 *Z* score	2.3	–	4.9	4.2	1.3
	rhGH dose	0.14	0.27	0.22^a^	0.13^a^	–
6	IGF-1 *Z* score	− 1.2	–	1.1	1.3	0.9
	rhGH dose	0.15	0.31	0.33	0.32	–

^a^Dose reduced due to high IGF-1 levels

^b^Dose reduced due to crying spells

### Safety

Recombinant human growth hormone was generally well tolerated, and there were no serious adverse events (Table 2). On average, participants experienced approximately five treatment emergent adverse events. One participant experienced gait changes, and rhGH was terminated early at week 11 out of an abundance of caution due to the risk of slipped capital femoral epiphysis. The participant was evaluated by their pediatrician, and no additional workup was deemed necessary; gait normalized within 2 days after stopping rhGH and without further sequelae. Another participant required dose reduction due to crying spells. Crying spells in all three participants were attributed to increased emotional lability. There were no clinically significant abnormalities on laboratory blood work.

**Table 2:** Adverse events

Adverse event	Number of participants
Increase in appetite	3
Gastroenteritis	3
Polyuria/nocturia	3
Crying spells	3
Runny nose/cough/sneezing	3
Decrease in appetite	1
Fever	3
Worsening repetitive behavior	2
Eye/ear infection	2
Diarrhea	1
Worsening hyperactivity	2
Sleep disturbance	1
Disruptive behavior	1
Bruising at injection site	1
Sweating of hands/feet	1
Limping/gait changes	1

### Efficacy

**Fig. 1:**
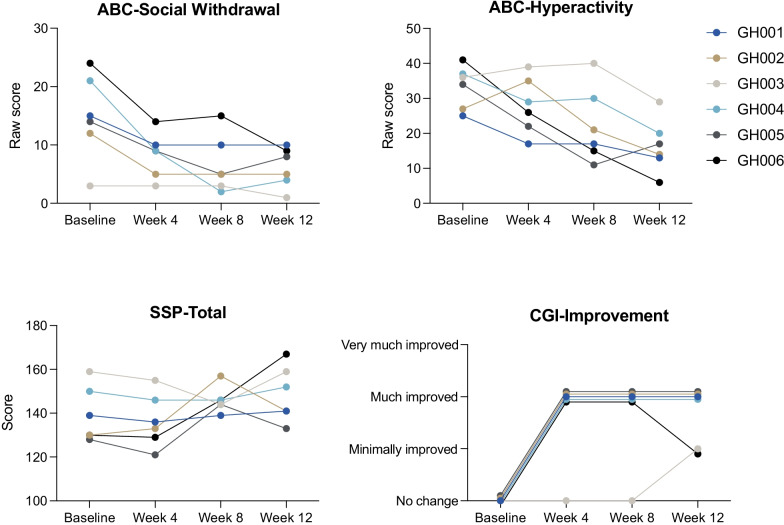
Domains of clinical improvement. Lower ABC scores indicate improved behavior, and higher SSP scores indicate improved behavior

There was an improvement in our primary clinical outcome, the ABC-SW subscale, between baseline and week 12 (*p* = 0.028) (Fig. 1). There was also an improvement in hyperactivity using the ABC hyperactivity subscale (*p* = 0.027), and in overall sensory symptoms as measured by the short sensory profile total score (*p* = 0.042). Overall, there was global improvement as measured by the CGI-I (*p* = 0.023). There were no significant changes in other clinical domains (Table 3).

**Table 3:** Summary statistics for clinical outcomes

Measure^a^	Variable	Baseline Mean (SD)	Week 12 Mean (SD)	*p* value	Wilcoxon *r* effect
ABC	Irritability	10.31 (7.6)	4.6 (2.3)	0.225	0.50
	Social withdrawal	14.8 (7.4)	6.2 (3.4)	0.028	0.90
	Stereotypy	8.8 (6.9)	5.1 (1.8)	0.249	0.47
	Hyperactivity	33.3 (6.2)	16.5 (7.7)	0.027	0.90
	Inappropriate speech	3.3 (4.3)	2.3 (3.8)	0.285	0.44
RBS-R	Stereotyped behavior	4.3 (1.7)	4.0 (2.4)	0.577	0.23
	Self-injurious behavior	0.7 (0.8)	1.3 (1.8)	0.157	0.58
	Compulsive behavior	2.8 (3.2)	1.3 (1.6)	0.083	0.71
	Ritualistic behavior	1.2 (1.2)	0.8 (1.2)	0.414	0.33
	Sameness behavior	2.3 (2.4)	2.0 (1.7)	0.680	0.17
	Restricted behavior	2.0 (2.2)	1.2 (1.5)	0.129	0.62
	Total	13.3 (6.1)	10.7 (3.1)	0.248	0.47
SSP	Tactile	31.5 (2.1)	31.7 (2.0)	1.00	0.00
	Taste/smell	18.2 (3.6)	19.4 (1.3)	0.317	0.41
	Movement	13.3 (1.6)	13.7 (1.8)	0.157	0.58
	Sensation	17.5 (4.8)	20.0 (2.4)	0.416	0.33
	Auditory	20.0 (1.9)	22.0 (2.4)	0.144	0.60
	Low energy/weak	16.5 (6.9)	20.5 (5.2)	0.141	0.60
	Visual/auditory	22.3 (2.3)	22.7 (1.5)	0.414	0.33
	Total	139.3	148.8	0.042	0.83
CGI	Improvement score	–	1.7 (0.5)	0.023	0.93

*ABC* Aberrant Behavior Checklist, *CGI* Clinical Global Impressions scale, *RBS-R* Repetitive Behavior Scales—Revised, *SSP* Short Sensory Profile

^a^For the ABC and RBS-R, lower scores indicate better performance; for the SSP and CGI-Improvement scale, higher scores indicate better performance

## Discussion

The results of this pilot open-label clinical trial demonstrate that standard clinical doses of rhGH increased levels of IGF-1 in children with PMS by at least 2SD from baseline for all participants; final levels of greater than or equal to 2SD were reached in all except one participant. Further, we show that rhGH was well tolerated without serious adverse events. As rhGH is already FDA-approved and established as safe in children with growth-related problems and in adults with growth hormone deficiency, these results provide preliminary evidence of safety in a new patient population without specific growth issues. rhGH treatment was also associated with clinical improvement that parallels the effects of IGF-1 on social withdrawal in this population. In addition, rhGH was associated with benefits in hyperactivity and sensory symptoms, all leading to global improvement based on the CGI-I. Studies of rhGH in PMS are ongoing using a randomized, placebo-controlled, crossover design. In addition, it will be critical to discover biomarkers to predict treatment response to rhGH in PMS, and potentially, within subgroups of ASD more broadly.

## Limitations

Results should be interpreted with caution given the small sample size and open-label design of the study.

## Conclusions

Taken together, these findings support the development of rhGH as treatment for children with PMS. Future studies of the effects of rhGH in PMS using an adequately powered placebo-controlled design are warranted.

## Data Availability

The datasets used and/or analyzed during the current study are available from the corresponding author on reasonable request.

## Abbreviations

ABC: Aberrant Behavior Checklist
AEs: Adverse events
ASD: Autism spectrum disorder
CGI-I: Clinical Global Impression—Improvement Scale
IGF-1: Insulin-like growth factor-1
PMS: Phelan–McDermid syndrome
RBS-R: Repetitive Behavior Scale—Revised
rhGH: Recombinant human growth hormone
SD: Standard deviation
SLAES: Systematic longitudinal adverse events scale
SSP: Short sensory profile

## References

[1] Bonaglia MC, Giorda R, Borgatti R, Felisari G, Gagliardi C, Selicorni A (2001). Disruption of the ProSAP2 gene in a t(12;22)(q24.1;q13.3) is associated with the 22q133 deletion syndrome. Am J Hum Genet.

[2] Wilson HL, Wong AC, Shaw SR, Tse WY, Stapleton GA, Phelan MC (2003). Molecular characterisation of the 22q13 deletion syndrome supports the role of haploinsufficiency of SHANK3/PROSAP2 in the major neurological symptoms. J Med Genet.

[3] Bonaglia MC, Giorda R, Mani E, Aceti G, Anderlid BM, Baroncini A (2006). Identification of a recurrent breakpoint within the SHANK3 gene in the 22q13.3 deletion syndrome. J Med Genet.

[4] Durand CM, Betancur C, Boeckers TM, Bockmann J, Chaste P, Fauchereau F (2007). Mutations in the gene encoding the synaptic scaffolding protein SHANK3 are associated with autism spectrum disorders. Nat Genet.

[5] Soorya L, Kolevzon A, Zweifach J, Lim T, Dobry Y, Schwartz L (2013). Prospective investigation of autism and genotype–phenotype correlations in 22q13 deletion syndrome and SHANK3 deficiency. Mol Autism.

[6] De Rubeis S, Siper PM, Durkin A, Weissman J, Muratet F, Halpern D (2018). Delineation of the genetic and clinical spectrum of Phelan–McDermid syndrome caused by SHANK3 point mutations. Mol Autism.

[7] Betancur C, Buxbaum JD (2013). SHANK3 haploinsufficiency: a “common” but underdiagnosed highly penetrant monogenic cause of autism spectrum disorders. Mol Autism.

[8] Boeckers TM (2006). The postsynaptic density. Cell Tissue Res.

[9] Bozdagi O, Tavassoli T, Buxbaum JD (2013). Insulin-like growth factor-1 rescues synaptic and motor deficits in a mouse model of autism and developmental delay. Mol Autism.

[10] Shcheglovitov A, Shcheglovitova O, Yazawa M, Portmann T, Shu R, Sebastiano V (2013). SHANK3 and IGF1 restore synaptic deficits in neurons from 22q13 deletion syndrome patients. Nature.

[11] Kolevzon A, Bush L, Wang AT, Halpern D, Frank Y, Grodberg D (2014). A pilot controlled trial of insulin-like growth factor-1 in children with Phelan–McDermid syndrome. Mol Autism.

[12] Marchetto MC, Carromeu C, Acab A, Yu D, Yeo GW, Mu Y (2010). A model for neural development and treatment of Rett syndrome using human induced pluripotent stem cells. Cell.

[13] Khwaja OS, Ho E, Barnes KV, O'Leary HM, Pereira LM, Finkelstein Y (2014). Safety, pharmacokinetics, and preliminary assessment of efficacy of mecasermin (recombinant human IGF-1) for the treatment of Rett syndrome. Proc Natl Acad Sci USA.

[14] Castro J, Garcia RI, Kwok S, Banerjee A, Petravicz J, Woodson J (2014). Functional recovery with recombinant human IGF1 treatment in a mouse model of Rett Syndrome. Proc Natl Acad Sci USA.

[15] Tropea D, Giacometti E, Wilson NR, Beard C, McCurry C, Fu DD (2009). Partial reversal of Rett syndrome-like symptoms in MeCP2 mutant mice. Proc Natl Acad Sci USA.

[16] Pini G, Congiu L, Benincasa A, DiMarco P, Bigoni S, Dyer AH (2016). Illness severity, social and cognitive ability, and EEG analysis of ten patients with Rett syndrome treated with mecasermin (recombinant human IGF-1). Autism Res Treat.

[17] Lopez-Lopez C, LeRoith D, Torres-Aleman I (2004). Insulin-like growth factor I is required for vessel remodeling in the adult brain. Proc Natl Acad Sci USA.

[18] O'Kusky JR, Ye P, D'Ercole AJ (2000). Insulin-like growth factor-I promotes neurogenesis and synaptogenesis in the hippocampal dentate Gyrus during postnatal development. J Neurosci.

[19] Costales J, Kolevzon A (2016). The therapeutic potential of insulin-like growth factor-1 in central nervous system disorders. Neurosci Biobehav Rev.

[20] Jorge AA, Grimberg A, Dattani MT, Baron J (2021). Disorders of childhood growth. Sperling pediatric endocrinology.

[21] Xie RJ, Li TX, Sun C, Cheng C, Zhao J, Xu H (2021). Correction to: a case report of Phelan–McDermid syndrome: preliminary results of the treatment with growth hormone therapy. Ital J Pediatr.

[22] Aman MG, Singh NN, Stewart AW, Field CJ (1985). Psychometric characteristics of the aberrant behavior checklist. Am J Ment Defic.

[23] Lam KS, Aman MG (2007). The repetitive behavior scale-revised: independent validation in individuals with autism spectrum disorders. J Autism Dev Disord.

[24] Dunn W (1999). Sensory profile.

[25] Guy W, ECDEU Assessment Manual for PR, Rockville M. US Department of Health, Education, and Welfare, Public Health Service, Alcohol, Drug Abuse, and Mental Health Administration, NIMH Psychopharmacology Research Branch, Division of Extramural Research Programs. 1976.

